# Attitudes and Practices of the Use of Third-Generation Cephalosporins among Medical Doctors Practicing in Cameroon

**DOI:** 10.1155/2023/8074413

**Published:** 2023-02-15

**Authors:** Elisabeth Zeuko'o Menkem, Astride Labo Nanfah, Tiku Takang, Lesley Ryan Awah, Kenneth Awah Achua, Stanley Ekane Akume, Fabrice Fekam Boyom

**Affiliations:** ^1^Department of Biomedical Sciences, Faculty of Health Sciences, University of Buea, Buea, Cameroon; ^2^Antimicrobial and Biocontrol Agents Unit, Laboratory for Phytobiochemistry and Medicinal Plants Studies, University of Yaounde 1, Yaoundé, Cameroon; ^3^Department of Clinical Studies, Faculty of Health Sciences, University of Buea, Buea, Cameroon; ^4^Department of Public Health, Faculty of Health Sciences, University of Buea, Buea, Cameroon

## Abstract

**Background:**

Third-generation cephalosporins (3GC) are among the most prescribed antibiotics worldwide. Antibiotic resistance, usually due to misuse and overuse, is a feared complication of public health concern. However, there are limited data in Cameroon concerning the knowledge and use of 3GC in our health services. The aim of this study was to assess the knowledge and use of 3GC among medical doctors in Cameroon and to generate baseline information for a wider scale research and policy implementation.

**Methods:**

This study was a cross-sectional study conducted among medical doctors practicing in Cameroon in general. Convenience sampling was used and the data were collected from both the online questionnaire and the review of files of patients admitted and discharged within the month of April 2021 and analysed with the use of IBM SPSS v25. *Results and Discussion*. A total of 52 respondents from the online questionnaire and 31 reviewed files were retained. Of the respondents, 27% were female and 73% were male. The mean age and years of experience were 29.6 ± 2.9 and 3.6 ± 2.1 years, respectively. Only 32.7% had correct knowledge of the number of generations of cephalosporins, and 48.1% had knowledge of the antimicrobial target. All medical doctors (MD) identified ceftriaxone as a 3GC, and it was the most commonly prescribed 3GC (71%). Most of the MD considered 3GC to be an efficient antibiotic. Just over half (54.7%) knew the correct posology of ceftriaxone. Only 17% and 9.4% knew the right posology for cefotaxime and ceftazidime, respectively, for the management of early-onset neonatal infection (EONNI). The misuse of 3GC was mostly attributed to nurses, MD, and poor institutional policies.

**Conclusion:**

There is average knowledge on 3GC among MD, with ceftriaxone being the most widely known and prescribed. Misuse is common among nurses and doctors. Poor institutional policies and limited laboratory capacities are to be blamed.

## 1. Introduction

Antibiotics play a paramount role in reducing the burden of infectious and communicable diseases worldwide [1]. However, cephalosporins belong to the beta lactam group of bactericidal antibiotics acting at the level of the bacterial cell wall, leading to osmotic lysis [2–5]. They are grouped into five generations based on the period of discovery and their antimicrobial properties [6]. Third-generation cephalosporins are broad-spectrum antibiotic agents with an activity against both Gram-negative and Gram-positive bacteria [7]. As one progresses from the first through the fifth generation, the potency of the bactericidal activity of cephalosporins decreases against Gram-positive organisms and increases against Gram-negative and beta lactamase-producing bacteria [8].

The empirical indications for the use of third-generation cephalosporins (3GC) include central nervous system (CNS) infections (such as meningitis, as they have the potential to cross the blood-brain barrier), genitourinary tract infections, bone and joint infections, community-acquired pneumonia, and skin and soft tissue infections. In the case of specific therapy, they are active against Gram-negative bacteria causing meningitis, Lyme disease, *Pseudomonas pneumonia*, sepsis of Gram-negative bacterial origin, streptococcal endocarditis, penicillinase-producing* Neisseria gonorrhea*, chancroid, and Gram-negative osteomyelitis [7].

3GC is most commonly used because of its activity against most Gram-negative organisms and its availability [9]. Six parenteral 3GC have been introduced in clinical use in the past 10 years. The 3 most frequently available 3GC agents are cefotaxime, ceftriaxone, and ceftazidime [10]. Among the 3GC, ceftriaxone is the most commonly prescribed drug because it has a high antibacterial potency, wide spectrum of activity, and low potential of toxicity. However, the global trend shows misuse of that drug [9].

A study conducted in Tikur revealed that the inappropriate use of ceftriaxone was very high in the medical and emergency wards of the Tikur Anbessa Specialized Hospital. This could lead to the emergence of resistant pathogens consequently leading to treatment failure and increase cost of the therapy [11]. Another study reported ceftriaxone being the most frequently prescribed drug with high cost in inappropriate antibiotic use being twice higher than patients with appropriate treatment [12].

From observations during clinical rotations, 3GC seems to be a commonly prescribed antibiotic in emergency and patient wards in teaching hospitals in the Southwest Region of Cameroon. Therefore, the aim of this study was to assess knowledge and use of 3GC among medical doctors practicing in Cameroon in general and to verify the actual practice in the Buea Regional Hospital in order to generate data that could be used to create awareness on the use of 3GC.

## 2. Materials and Methods

### 2.1. Study Area

This study was conducted among medical doctors practicing in Cameroon in general. Most medical doctors practicing in Cameroon are graduates from one of the six faculties of medicine recognized by the state.

Part of the study was conducted at the internal medicine ward of Buea Regional Hospital. Buea Regional Hospital is a teaching hospital that serve as a referral hospital for district hospitals in the Southwest Region and its environs. The range of specialties included are internal medicine (neurology, nephrology, and cardiology), pediatrics, surgery, gynecology, obstetrics, and an outpatient clinic.

### 2.2. Study Design and Study Period

The study was a cross-sectional study with 02 forms of data collected:

- The primary data was collected through an online questionnaire designed with the use of Google Forms and shared through social media to medical doctors practicing in Cameroon. The questionnaire was circulated for only three working days.
- The secondary data was collected by reviewing patient's records in medical wards admitted and discharged within the month of April 2021 in the internal medicine ward at the Buea Regional Hospital. This study was conducted from 17th to 29th May, 2021.

### 2.3. Study Population

Medical doctors practicing in Cameroon. All patients admitted in the medical wards within the month of April 2021 and were present between 17th and 29th May, 2021.

#### 2.3.1. Inclusion Criteria

The inclusion criteria were as follows:

- All medical doctors practicing in Cameroon
- All records of patients treated with 3GC

#### 2.3.2. Exclusion Criteria

The exclusion criteria were as follows:

- Records of patients with incomplete data
- Medical doctors who did not give their consent
- Questionnaires with incomplete information
- Responses that came after 03 days of collection were not included in the analysis.

### 2.4. Sample Size

The patient's records in medical wards admitted and discharged within the month of April 2021 in the internal medicine ward at the Buea Regional Hospital during the study period (17th to 29th May, 2021). All doctors, available to answer the questionnaire either online or physically, were considered.

### 2.5. Data Collection Instruments and Data Collection Procedure

The appropriateness of ceftriaxone, cefotaxime, and ceftazidime utilization was evaluated using a standard treatment protocol, which was developed after a thorough literature review regarding the rational use of these 3GCs. Different studies, including the Stanford antibiotic guidelines, the National Institute of Health (NIH) journal, and the World Health Organization (WHO) guidelines, were used. The appropriateness of 3GC was determined based on the indication and the daily dose needed. A data abstract guide was prepared from different guidelines and research articles to include information about sociodemographic characteristics, clinical characteristics, common indications, dose, frequency of use, and concomitant drugs used with 3GC.

The data were collected from both the online questionnaire and a review of the files of patients admitted and discharged within the month of April 2021.

The data collected from the online questionnaire included three sections: the first section included demography, age, sex, specialty, and years of experience; the second section assessed the awareness and knowledge of the medical doctors on 3GC; and the third section included information regarding the use of ceftriaxone, cefotaxime, and ceftazidime.

The data collected from patients' reports were more focused on the posology and indications of ceftriaxone and whether or not culturing of microorganisms was performed. This information was intended to compare the link between what is reported from the online questionnaire and what is practiced.

### 2.6. Data Analysis

The data collected for both online and file review were entered into separate Excel sheets. Data were cleaned, coded and then exported for analysis using the Statistical Package for the Social Sciences (SPSS v25.0) software version 25.0 for Windows. The mean, standard deviation, maximum, and minimum values were computed for continuous data (age and years of experience), frequencies and proportions were used to analyse categorical data.

The questions with multiple answers and open questions were analysed manually by simple counts and grouping.

### 2.7. Ethical Consideration

The study was approved by the Faculty of Health Sciences, University of Buea, ref 2021/303/UB/FHS. Also, authorization was obtained from Buea Regional Hospital Review Board of 24 May, 2021.

To ensure anonymity, codes were used for the online questionnaire as well as the files reviewed. Only generic names of the drugs were used. No identifying information about the patients was collected.

## 3. Results and Discussion

### 3.1. Results

54 responses were collected from the online questionnaire, of which 2 were excluded because they did not meet the inclusion criteria.

Seventy (70) files were reviewed, and 31 were retained as the patients received ceftriaxone during their hospital stay.

#### 3.1.1. Demographic Data

Of the 52 respondents, 71.7% (38) were male. The mean age of the respondents was 29.62 ± 2.91 years, with a minimum age of 24 years and a maximum of 38 years. In addition, the mean year of experience in the field was 3.59 ± 2.07 years, with a minimum of 1 year and a maximum of 10 years. Most of the respondents (81.1%) were general practitioners.

**Figure 1:**
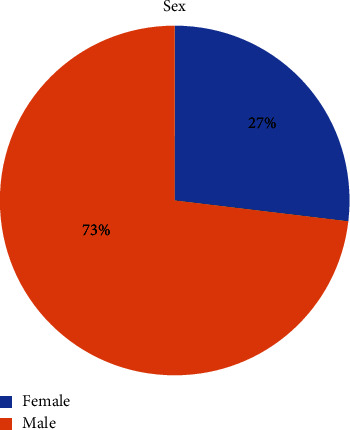
Sex distribution of respondents.

More male practitioners participated in the study, as shown in Figure 1.

#### 3.1.2. The Level of Knowledge

A total of 92.3% (48) of the participants chose beta lactams as the correct antibiotic class of cephalosporins. Only 32.7% (17) had correct knowledge about the number of generations of cephalosporins. Moreover, 84.6% (44) recognized that cephalosporins inhibit bacterial cell walls. A total of 48.1% (25) were aware of the type of bacteria targeted by 3GC (that is, more Gram-negative than Gram-positive).

Fifty percent were informed of the use of 3GC as first-line antibiotics. A total of 55.8% (29) identified that the disulfuram reaction was a side effect of cephalosporins. Only 17.3% (9) had correct knowledge of the half-life of 3GC, while 86.5% (45) knew the route of elimination (Table 1).

All the respondents identified ceftriaxone, 52 (100%) as a 3GC, and the least identified 3GCs were cefdodoxime 1 (1.9%) and ceftibuten 1 (1.9%), as shown in Table 1.

#### 3.1.3. The Practical Use of 3GC among Medical Doctors

From the study, ceftriaxone was the second most commonly prescribed antibiotic among practicing medical doctors in Cameroon, as shown in Table 2.

Of the 52 respondents, 71.2% (37) reported having prescribed ceftriaxone at least once in their practice, while the rest had yet to prescribe ceftriaxone.

Moreover, 84.6% considered 3GC efficient, and 7.7% could not highlight any efficiency based on their experience.

#### 3.1.4. The Posology of Ceftriaxone in Adult Medicine

The results of the posology of ceftriaxone are shown in Table 3. It was appropriate in 51.9% (27) of cases, with 48.1% (25) of the cases being inappropriate for the management of adult bacterial meningitis. In addition, it was appropriate for the management of adult acute pyelonephritis in 84.6% (44) of the cases and inappropriate in 11.5% (6) of the cases, with 3.85% (2) missing data. Moreover, ceftriaxone posology for the management of adult sepsis was appropriate in 70. 6% (36) of the cases and inappropriate in 29. 4% (15) of the cases.

#### 3.1.5. Posology of 3GC in the Management of Early Onset Neonatal Infection

The posology of cefotaxime in the management of early-onset neonatal infection (EONNI) was appropriate in 17% (9) of the cases and inappropriate in 66% (35) of the cases, with 17% (9) having no idea. In addition, ceftriaxone was appropriately prescribed in 54.7% (29) of the cases and inappropriately in 13.2% (7) of the cases, and 32.1% (16) had no idea. Ceftazidime was appropriately prescribed in 9.4% (5) of cases and was inappropriate in 45.3% (24) of the cases, with 45.3% (24) having no idea (Table 4).

#### 3.1.6. The Misuse of 3GC

**Figure 2:**
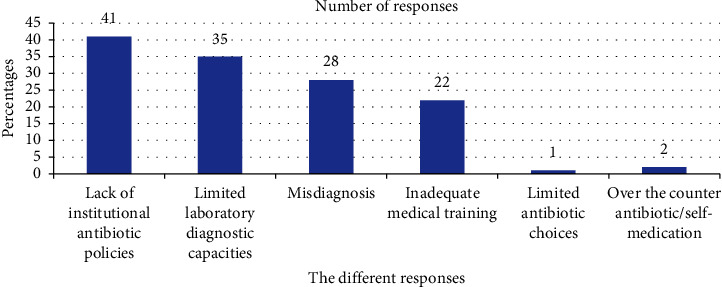
Assessment of possible causes of misuse.

**Figure 3:**
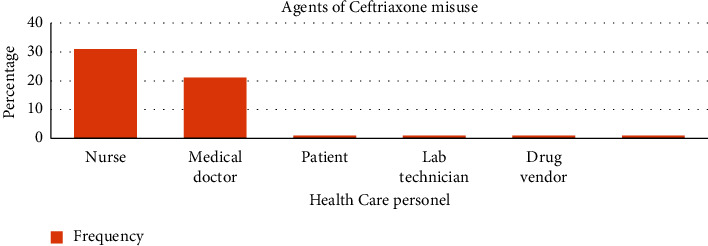
Report of ceftriaxone misuse.

A total of 94.2% of respondents reported having encountered misuse of ceftriaxone. The reasons given for the misuse were the lack of institutional policies 78.8% (41) and limited laboratory diagnostic capacities 67. 33% (35) (Table 5 and Figure 2). A total of 59.6% (31) indicated that misuse was performed by nurses, and up to 40.4% (21) indicated misuse was performed by medical doctors (Figure 3).

#### 3.1.7. The Practical Use of 3GC from File Review

Seventy files were reviewed, with ceftriaxone being prescribed in 31 files. The pathologies for which it was prescribed were CNS infections (meningitis/encephalitis) 25.81% (8), sepsis 19.35% (6), and malaria 9.68% (3) (Table 6).

The two 3GC frequently prescribed were ceftriaxone for intravenous administration (100%), and cefixime was used orally as relay in 38.7% (12) out of 31 patients. There was an inappropriate use of ceftriaxone in 51.61% of these files.

### 3.2. Discussion

This study revealed that ceftriaxone was the most commonly used 3GC among medical doctors in Cameroon. It was also the only intravenous 3GC used in the medical ward of Buea Regional Hospital. However, it is the best way to deliver a dose rapidly and accurately, as the drug enters directly into systemic circulation without the delay associated to absorption processes, achieving its therapeutic effect faster than by any other route. Also, this route presents a bioavailability of 100%, since the pharmaceutical active ingredient usually reaches the site of action without suffering alterations due to presystemic effects [16–19]. Though this route is very efficient, it needs the application of the right dose of the drug due to right prescription, for low or high dose of the drug may lead to many consequences such as antibiotic resistance, low efficacy of the drug, long stay in the hospital, and long treatment leading to high cost and possible high mortality rate [20–22]. The result of our study showed that ceftriaxone was used as empirical treatment in 100% of patients, which is higher than 79.5% in a study performed by Sileshi et al. [11], 83.7% in a study conducted by Ayele et al. [15], and 82% in a study conducted by Pereira et al. [12]. This difference could be explained by the fact that their studies were carried out in tertiary facilities with improved laboratory diagnostic potentials.

The most common indications of ceftriaxone were central nervous system (CNS) infection (25.8%), followed by sepsis and malaria (Table 6). This is different from the study conducted in Gondar University Referral Hospital (GURH), where the most common indication was RTI (29.3%) followed by CNS infection (24.1%) [15], RTI (35.4%) in General Hospital, port of Spain [12], and pneumonia (35%) in Addis Ababa [11]. This could be explained by the fact that the protocols used in Buea Regional Hospital (BRH) for the management of RTI were first penicillin and macrolides, not 3GC.

In this study, the culture of microorganisms and the sensitivity test were not performed in all the cases (100%) of 3GC prescription compared to the GURH study where it was not performed in 85.5% of cases [15]. The possible reasons could be the relatively high cost of culture and sensitivity, the lack of laboratory capacity for culture in many facilities as reported by 41 of the 52 (78.8%) respondents, and the fact that culture takes a minimum of three days for results to be available, which compromises the health status of the patients. Due to this, most physicians prefer treating the patient empirically rather than sending them for culture and sensitivity in the laboratory.

Only 9.4% of the respondents could give the appropriate posology of ceftazidime for the management of early-onset neonatal infection (EONNI) (Table 4), which is very low compared to the study conducted in a tertiary hospital in Ethiopia by Gebremichael et al., where ceftazidime was appropriately used in 29.2% of the cases [23]. This difference could be attributed to the fact that the criteria to determine the appropriate use of ceftazidime in that study were many: indication and posology, while in our case, the appropriateness was based on a single posology.

A total of 51.61% of ceftriaxone used in the medical ward of the BRH was found to be inappropriate. The results are lower than the study done in GURH (80.2%) [15] and Tikur Anbessa in Addis Ababa (87.9%) [11]. The difference could be attributed to the differences in study designs and the rigorous criteria used (indication, daily dose administration, treatment duration, presence or absence of culture results, route of administration, and duration of hospital stay) to assess appropriateness, whereas this study focused on the indication and daily dose. In addition, a smaller sample size was used.

The rate of appropriateness of ceftriaxone used with the online questionnaire (51.8% for bacterial meningitis, 84.6% for acute pyelonephritis, and 67.9% for sepsis) was higher than the rate obtained from the files (48.38%). This difference could be explained by the fact that the management of the patient in the ward is multifactorial and depends on his/her comorbidities, while the questions asked on the posology by the online questionnaire were straight forward and did not consider any patient factor. In addition, one could suggest that since the online questionnaire was not time restricted, some respondents could have cross-checked the posology.

All this information indicates that there is a need for a stewardship assessment to be implemented and performed in line with hospital outcome indicators [24]. Also, as one of the outcome indicators, prolonged length of stay is directly linked to higher hospital costs [25]. More so, the prolonged antibiotic exposure, in fact, is associated with multiple drawbacks like the emergence of antibiotic resistance [13, 25].

## 4. Limitations

This study was carried out over a limited period. This did not give time to comprehensively assess all patients' records in the various wards of the hospital or to develop a well-structured questionnaire with a timer that could have reached more MD. In addition, the time factor affected our literature search.

The online questionnaires were not time restricted; this could give a room to response bias because some respondents could cross-check responses from other sources before filling. To limit this, we circulated the questionnaire for only three working days. Responses that came after that were not included in the analysis. No resource on a similar subject was found in the literature on Cameroon.

## 5. Conclusion, Recommendations, and Limitations

### 5.1. Conclusion

The present study revealed that medical doctors in Cameroon have an average knowledge of cephalosporins and 3GC in particular. However, most MDs were more informed about ceftriaxone as a 3GC, and it was the most widely prescribed. There is also a high rate of inappropriate use of ceftriaxone, which may potentially lead to the emergence of drug-resistant microorganisms and ultimately expose patients to treatment failure and a higher cost of therapy. Again, empirical treatment with ceftriaxone was found to be related to inappropriate use.

The majority of the MD reported a lack of institutional antibiotic policies and limited laboratory capacity to be the cause of 3GC misuse in health facilities. The high rate of ceftriaxone misuse was performed by nurses, followed by medical doctors.

### 5.2. Recommendations

To improve the knowledge and better practice of antibiotic use and 3GC in particular, regular, continuous medical education sessions should be organized at the institutional level and at the national level. In addition, strong institutional policies and regularly updated antibiotic policies need to be developed. The prescription of antibiotics by nurses should be highly regulated.

The laboratories (both public and private) should be equipped with the capacity to perform the culture of microorganisms and sensitivity when needed.

## Figures and Tables

**Table 1:** Assessment of the knowledge of medical doctors on cephalosporins and 3GC.

	Knowledge assessment	Frequency	Percentage (%)
Class of cephalosporin	Correct	48	92.3
	Incorrect	4	7.7

Number of generations of cephalosporins	Correct	17	32.7
	Incorrect	35	67.3

Mechanism of action of 3GC	Correct	44	84.6
	Incorrect	8	15.4

Bacteria target of 3GC	Correct	25	48.1
	Incorrect	27	51.9

Half-life of 3GC	Correct	9	17.3
	Incorrect	43	82.7

Route of elimination	Correct	45	86.5
	Incorrect	7	13.5

Side effects	Correct	29	55.8
	Incorrect	23	44.2

**Table 2:** Antibiotics frequently prescribed by medical doctors.

Antibiotic most prescribed	Frequency	Percentage (%)
Amoxicillin	24	46.15
Ceftriaxone	18	34.61
Cefixime	1	1.92
Metronidazole	1	1.92
Ofloxacin	1	1.92
Ciprofloxacin	1	1.92
Azithromycin	1	1.92
Unknown	5	9.61

**Table 3:** Assessment of the posology of ceftriaxone in adult medicine.

Indication	Posology	Frequency	Percentage (%)	*P* value
Bacterial meningitis	Correct	27	**51.9**	*P* < 0.05
	Incorrect	25	48.1	

Acute pyelonephritis	Correct	44	**84.6**	*P* < 0.01
	Incorrect	6	11.5	
	No response	2	3.8	

Sepsis	Correct	36	**70.6**	*P* < 0.01
	Incorrect	15	29.4	

The bold values indicate statistical significant results posology of ceftriaxone in the treatment of bacterial meningitis, acute pyelonephritis, and sepsis being respected.

**Table 4:** Assessment of knowledge on posology of some 3GC in EONNI.

Indication	3GC	Posology	Frequency	Percentage (%)
Early-onset neonatal sepsis (EONNI)	Ceftriaxone	Appropriate	**29**	**54.7**
		Inappropriate	7	13.2
		No response	**16**	**32.1**
	Cefotaxime	Appropriate	**9**	**17.0**
		Inappropriate	35	66.0
		No response	**9**	**17.0**
	Ceftazidime	Appropriate	**5**	**9.4**
		Inappropriate	24	45.3
		No response	**24**	**45.3**

The bold values indicate that for ceftriaxone row *P* < 0.05, indicating that the posology was appropriate. For Cefotaxime, *P* = 0.05, with the inappropriate posology being observed. For ceftazidime, *P* = 0.05.

**Table 5:** Assessment of causes of ceftriaxone misuse.

Reason of misuse	Frequency of response	Percentage (%)
Lack of institutional antibiotic policies	41	78.8
Limited laboratory diagnostic capacities	35	67.33
Misdiagnosis	28	53.8
Inadequate medical training	22	42.3
Limited antibiotic choices	1	1.9
Over the counter antibiotic/self-medication	2	3.8

**Table 6:** Proportions of the indications of ceftriaxone prescribed from the file review.

Indication	Number of prescriptions	Percentage (%)
Meningitis/encephalitis	8	25.8
Sepsis	6	19.35
Malaria	3	9.68
Enterocolitis	1	3.22
Dementia	1	3.22
Pericarditis	1	3.22
Atypical pneumonia	1	3.22
Acute pyelonephritis	1	3.22
Cholecystitis	1	3.22
CVA	2	6.44
CKD/AKI	2	6.44
Cellulitis/leg ulcer	2	6.44
Cerebral toxoplasmosis	1	3.22
CHF	1	3.22

CHF: congestive heart failure; CKD/AKI: chronic kidney disease/acute kidney disease; CVA: cerebrovascular accidents.

## Data Availability

The data used to support the findings of the study are available from the corresponding author upon request.
